# Sunscreen Formula of FeO(OH)·H_2_O/TiO_2_ With Spectral Selectivity to Enhance Collagen Biosynthesis via Fibroblast Test

**DOI:** 10.1111/jocd.70060

**Published:** 2025-02-13

**Authors:** Xun Zhang, Ximing Wen, Yuling Wang, Fangru Jiang, Yiting Chen, Zhe Liu

**Affiliations:** ^1^ Bloomage Biotech Co. Ltd. Jinan Shandong China

**Keywords:** collagen, photoaging, skin health, spectral selectivity, sunscreen

## Abstract

**Objective:**

To develop a sunscreen formula with spectral selectivity that filters harmful light (280–550 nm) while allowing beneficial light (550–760 nm) to permeate and thus to boost collagen generation.

**Methods:**

A variety of sunscreen filters and their combinations were tested for transmittance spectrum. The spectral selectivity was quantified by Selection Index (SI), and the optimal formula was identified. Then, human dermal fibroblasts (HDFs) were subjected to simulated sunlight exposure with the application of this formula. The cell viability and collagen levels were measured post‐exposure.

**Results:**

The combination of TiO_2_ and FeO(OH)·H_2_O displays spectral selectivity and reaches the optimal SI value at the mixing ratio of 64:36. This mixture, when formulated with traditional UV filters, significantly elevates the level of Collagen I.

**Conclusion:**

This work uncovers the influence of spectral selectivity on the enhancement of sunscreen performance and proposes a filter combination with spectral selectivity. This formula, when integrated with conventional UV sunscreens, allows for the beneficial effects of sunlight to be more pronounced. This discovery may provide fresh insights for the design of future sunscreen products.

## 1 Background

Excessive exposure to sunlight would cause severe skin damage, including sunburn, photoaging, and even skin cancer [1, 2, 3]. Specifically, UVB (280–320 nm) is primarily implicated in sunburn and DNA damage [4, 5]. UVA (320–400 nm) significantly contributes to photoaging [6, 7]. High‐energy visible light (HEV, 400–550 nm) has effects akin to UVA, as it can also penetrate dermal layer to cause oxidative stress [8, 9]. Recent research indicates that high doses and prolonged exposure to HEV may enhance the production of ROS (reactive oxygen species) and RNS (reactive nitrogen species), leading to cellular damage and photoaging [10].

While, sunlight exposure regulates a variety of endogenous processes, such as circadian rhythms and core body temperature [11]. Moreover, certain wavelengths in the visible light spectrum could deliver benefits to the skin [12]. Weiss [13] utilized 590 nm irradiation to treat 90 patients with photoaged skin, revealing significant skin improvements, including enhanced smoothness, reduced periorbital wrinkles, and lessened erythema and hyperpigmentation. Barolet [14] reported that yellow light at 660 nm can positively influence the skin's appearance by reducing matrix metalloproteinase‐1 (MMP‐1) expression and enhancing collagen production. Wunsch [15] indicated that light with 570–760 nm wavelength exhibits a positive effect on skin regeneration and increases collagen synthesis.

The pursuit of effective sun protection is a long‐standing human endeavor, with records indicating that ancient Egyptians, as early as the 15th century BC, used natural substances like rice bran and jasmine against the sun's harmful effects. This use of plant‐based sunscreens persisted, with oils from olives and walnuts later recognized for their sun‐blocking properties. The 20th century ushered in a scientific approach with the introduction of inorganic filters, specifically zinc oxide (ZnO) and titanium dioxide (TiO_2_), which physically shield against UV rays [16]. These substances, however, necessitate high doses for adequate protection. The 1940s marked a key advancement with the creation of para‐aminobenzoic acid (PABA), the first chemical sunscreen, which efficiently absorbed UVB radiation at low concentrations [17]. Subsequently, numerous organic sunscreens, such as homosalate, octocrylene (OCT), benzophenone, and avobenzone (AVO) emerged [18]. Moreover, some broad‐spectrum filters that can absorb both UVA and UVB rays were invented, an example being Bisoctrizole.

Yet, the challenge of selectively filtering sunlight remains. This study seeks to address this by developing a formula that filters harmful 280–550 nm light while allowing beneficial 550–760 nm wavelengths. The efficacy of this formula was evaluated via cellular experiments in this work.

## 2 Experiments

### 2.1 Materials

The following sunscreen filters were employed: ZnO (Co‐fun Biotech, Zn‐5000), TiO_2_ (Co‐fun Biotech, Uni‐TW‐Si02GL), Fe_2_O_3_ (Co‐fun Biotech, RP‐29SG), FeO(OH)·H_2_O (Co‐fun Biotech, YP‐75SG), and Fe_3_O_4_ (Co‐fun Biotech, BP‐50SG), Octocrylene (DSM, PARSOL 340), Avobenzone (DSM, PARSOL 1789), and Methylene Bis‐Benzotriazolyl Tetramethylbutylphenol (DSM, PARSOL MAX), all of which were with > 99% purity. Diethylhexyl carbonate, provided by Evonik Industries AG, was used as the dispersant in spectrum measurements. A commercial sunscreen (KE'AN REFRESH, men's light physical sunscreen cream, LOT#20131329) was used as benchmark.

Human dermal fibroblasts (HDFs), DMEM medium (dulbecco's modified eagle medium, 31600‐034), MTT solution (3‐(4,5)‐dimethylthiahiazo (‐z‐y1)‐3,5‐di‐phenytetrazoliumromide, M158055), and PBS medium (phosphate buffered saline, P1010) were utilized for cell tests, and they were supplied by Guangdong Biocell Biotechnology Co. LTD. Type I collagen antibody (Proteintech, 66761‐1‐Ig) and staining solution for live cells (Hoechst, 33342) were employed for immunofluorescence tests, and the paraformaldehyde (Biosharp, BL539A) were used for cell fixing.

### 2.2 Spectrum Measurement

The light transmittance spectrum was measured by UV–Vis Spectrophotometer (Mettler Toledo, UV5 nano), and the scanning step was set as 0.2 nm. Firstly, individual filters were dispersed in diethylhexyl carbonate at 0.05 mol/L, respectively. A sonicating device (Ningbo Scientz Biotechnology, SB‐5200DTD) was employed to homogenize the suspensions for 10 min. Then, the samples were filled in quartz cells (Tansoole, TS010‐015) to measure the transmission spectrum. After that, the selected filters were formulated for further measurements, as shown in Table 1.

**TABLE 1:** Sunscreen formula in spectrum measurements.

Case	Filter combination (Wt %)	Total concentration of the filters (*10^−4^ mol/L, diethylhexyl carbonate as the solvent)
UV‐1	87% TiO_2_ + 13% FeO(OH)·H_2_O	5.0
UV‐2	75% TiO_2_ + 25% FeO(OH)·H_2_O	5.0
UV‐3	64% TiO_2_ + 36% FeO(OH)·H_2_O	5.0
UV‐4	53% TiO_2_ + 47% FeO(OH)·H_2_O	5.0
UV‐5	43% TiO_2_ + 57% FeO(OH)·H_2_O	5.0
UV‐6	33% TiO_2_ + 67% FeO(OH)·H_2_O	5.0
UV‐7	24% TiO_2_ + 76% FeO(OH)·H_2_O	5.0
UV‐8	16% TiO_2_ + 84% FeO(OH)·H_2_O	5.0
UV‐9	8% TiO_2_ + 92% FeO(OH)·H_2_O	5.0
UV‐10	64% TiO_2_ + 36% FeO(OH)·H_2_O	25.0
UV‐11	64% TiO_2_ + 36% FeO(OH)·H_2_O	1.0
UV‐12	62.7% TiO_2_ + 36% FeO(OH)·H_2_O + 1.3% Octocrylene	5.0

The definition of light transmittance (T), as shown in Equation (1), quantifies the proportion of transmitted light intensity relative to that of the incident light. A lower T value signifies superior sunscreen blocking efficacy.

(1) $$T=I_{t}/I_{0}$$

where,

*I*_t_: the intensity of the transmitted light;

*I*_0_: the intensity of the incident light.

The Sun Protection Factor (SPF) can be deduced from light transmission data, using analytical tools like SUNSCREEN OPTIMIZER [19] or the empirical equation presented in Equation (2) [20]. Essentially, the SPF value inversely correlates with the mean transmittance within the 280–320 nm wavelength range (*T*_*mean*_). For instance, an SPF 30 product would theoretically permit only 3.3% of UVB rays to penetrate the sunscreen barrier to the skin. However, it is crucial to acknowledge that this theoretical SPF might vary from actual clinical outcomes due to influencing factors such as the thickness of application, skin type variations, and environmental conditions during testing.

(2) $$\mathrm{SPF}=1/{\text{T}}_{\mathrm{mean}}$$

Although the SPF value offers a metric for a sunscreen's defense against UVB radiation, it does not fully encapsulate the spectrum‐wide filtering properties of the product. To address this gap, our study proposes the Selection Index (SI), designed to complement SPF by evaluating sunscreen performance in terms of spectral selectivity. The SI measures the differential transmittance of beneficial light in the 550–760 nm wavelength range and harmful light in the 280–550 nm range, as detailed in Equation (3). This differential is then normalized to the cumulative light transmittance between 280 and 760 nm. Therefore, an increase in beneficial light transmittance or a decrease in harmful light transmittance will manifest as an upward shift in the SI value.

(3) $$\mathrm{SI}=\frac{1}{\sigma }\left(\int_{550}^{760}T\left(\lambda \right)\mathrm{d\lambda}-\int_{280}^{550}T\left(\lambda \right)\mathrm{d\lambda}\right),\;\sigma =\int_{280}^{760}T\left(\lambda \right)\mathrm{d\lambda}$$

where,

*λ*: light wavelength;

*T*(*λ*): light transmittance at the wavelength of λ nm;

*σ*: total light transmittance in the range of 280–760 nm.

### 2.3 Cell Test

The test design is outlined in Table 2 and includes a negative control (NC), blank control (BC), and test legs (Cell‐1 to ‐9). Initially. HDFs were seeded in well plates and cultured in DMEM medium overnight at 37°C, under a 5% CO_2_ environment. Following that, transparent quartz covers were laid atop the well plates, and the sunscreen formulas were uniformly applied on these covers at a quantity of 9.2 mg/cm^2^. Then, the cells were subjected to a light source that mimics sunlight (280–750 nm sunlight simulator, Dr. Hönle GmbH). To be noted, the NC leg was not filtered by sunscreen formulas, and the BC leg was not exposed to the light source.

**TABLE 2:** Cell test design.

Case	Sunscreen formula applied on the optical filters (Wt %)	Irradiation wavelength (nm)	Irradiation intensity (J/cm^2^)
BC	/	/	/
NC‐1	100% Ethylhexyl Carbonate	280–760	5
NC‐2	100% Ethylhexyl Carbonate	280–760	10
NC‐3	100% Ethylhexyl Carbonate	280–760	20
Cell‐1	5% OCT + 5% Avo + 10% MBBT +80% Ethylhexyl Carbonate	280–760	10
Cell‐2	10% OCT + 10% Avo + 20% MBBT +60% Ethylhexyl Carbonate	280–760	10
Cell‐3	5% OCT + 5% Avo + 10% MBBT +1.6% TiO_2_ + 0.9% FeO(OH)·H_2_O + 77.5% Ethylhexyl Carbonate	280–760	10
Cell‐4	5% OCT + 5% Avo + 10% MBBT +3.2% TiO_2_ + 1.8% FeO(OH)·H_2_O + 75% Ethylhexyl Carbonate	280–760	10
Cell‐5	5% OCT + 5% Avo + 10% MBBT +2.5% ZnO + 77.5% Ethylhexyl Carbonate	280–760	10
Cell‐6	OCT 5% + Avo 5% + MBBT 10% + 2.5% Fe_3_O_4_ + 77.5% Ethylhexyl Carbonate	280–760	10
Cell‐7	5% OCT + 5% Avo + 10% MBBT +5% ZnO + 75% Ethylhexyl Carbonate	280–760	10
Cell‐8	5% OCT + 5% Avo + MBBT 10% + 5% Fe_3_O_4_ + 75% Ethylhexyl Carbonate	280–760	10
Cell‐9	Commercial sunscreen containing 25% ZnO	280–760	10

Abbreviations: Avo represents Avobenzone; MBBT represents 2,2′‐Methylene‐bis‐(6‐(2H‐benzotriazol‐2‐yl)‐4‐(1,1,3,3‐tetramethylbutyl)phenol); OCT represents octocrylene.

After treatments, the cells were further cultured for 24 h. Then, the cell viability was evaluated by using MTT assay. Meanwhile, the levels of collagen I were determined by immunofluorescence. The integrated fluorescence density of each case was calculated and then normalized by the result of BC leg. The normalized fluorescence signal (NFS) was used to present the relative contents of collagen I.

Each case was tested with six replicates, and the results were compared by a double‐tailed *t*‐test. The level of significance when compared to the BC group is denoted as “#”. Here, a single “#” represents “*p*‐value < 0.05,” and “##” signifies “*p*‐value < 0.01.” Conversely, when compared to the NC group, the level of significance is indicated by “*”.

In this case, a single “*” corresponds to “*p*‐value < 0.05,” and “**” stands for “*p*‐value < 0.01.”

## 3 Results

### 3.1 Optimal Sunscreen Formula

**FIGURE 1:**
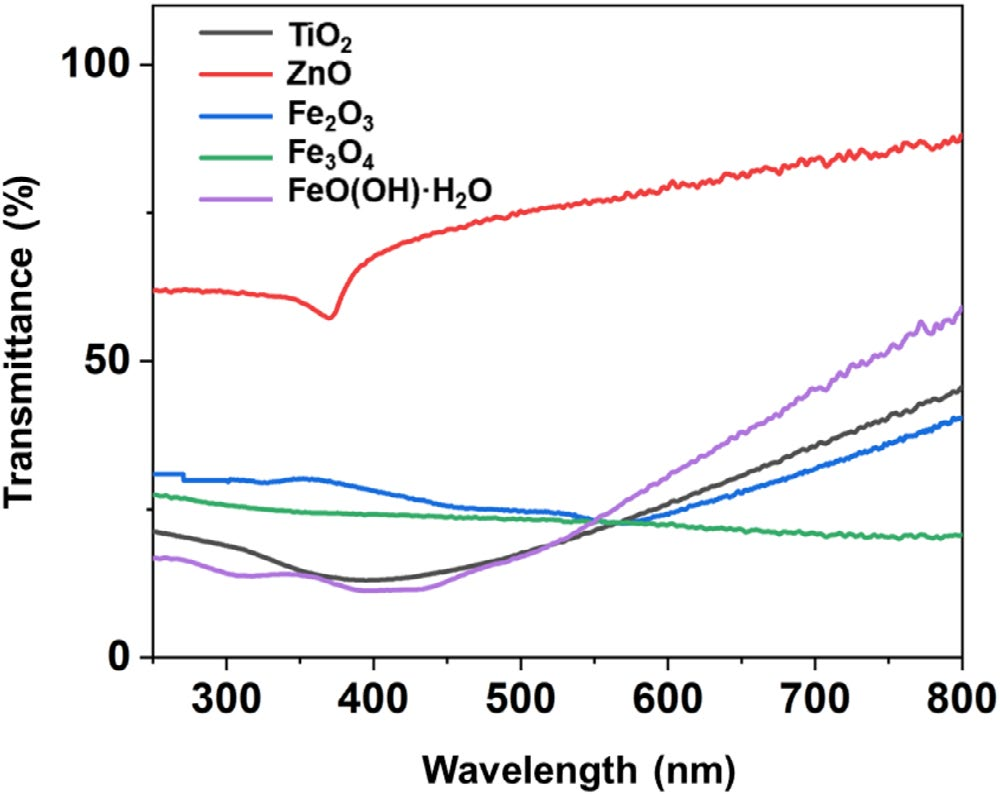
UV–Vis transmittance spectrum of individual inorganic filters.

Filter candidates for the desired sunscreen formula were selected based on transmittance analysis, as depicted in Figure 1. TiO_2_ and FeO(OH)·H_2_O are the lead options thanks to their minimal transmittance in the harmful wavelength region (280–550 nm) and enhanced transmittance in the beneficial range (550–750 nm). Fe_3_O_4_, on the other hand, was characterized by a uniform transmittance profile throughout the 280–760 nm spectrum. Although ZnO exhibits a trend of increased transmittance at higher wavelengths, this trend begins to manifest at the wavelength of 400 nm, resulting in increased transmission of some harmful light. Fe_2_O_3_ was deemed less suitable, as it allowed excessive transmittance in the higher energy wavelengths while displaying weak transmittance in the lower energy range.

**FIGURE 2:**
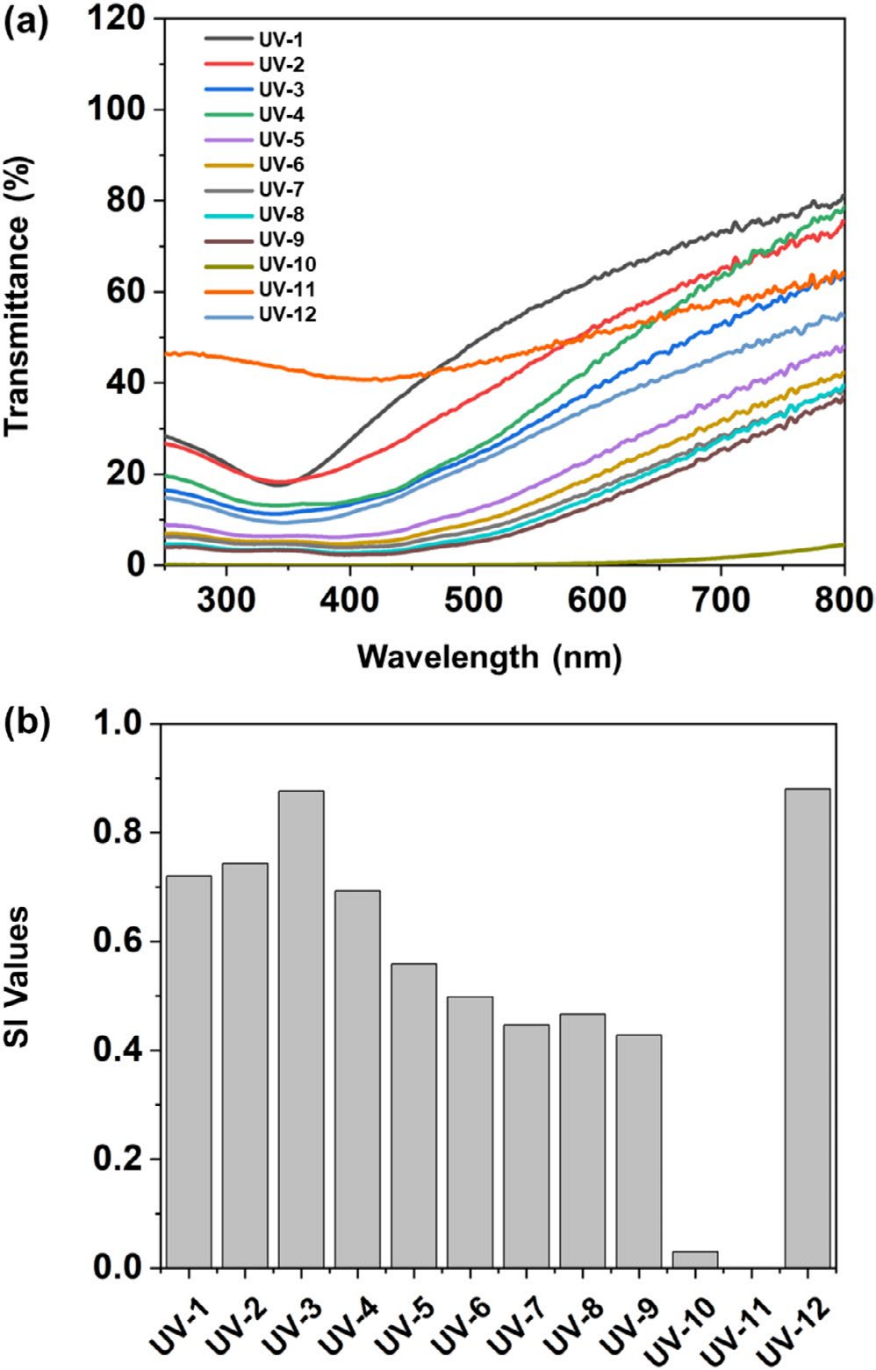
(a) UV–Vis transmittance spectrum, (b) SI values of different sunscreen formulas UV‐1 to UV‐12.

Subsequently, a series of TiO_2_ and FeO(OH)·H_2_O mixtures at varying ratios were prepared to optimize the SI value. The corresponding transmittance curves and SI values are illustrated in Figure 2. Upon reviewing the SI values from UV‐1 to UV‐9, a clear pattern emerged, an initial increase in the SI value followed by a decrease as the proportion of FeO(OH)·H_2_O was raised, culminating at the peak with UV‐3, where the mass ratio was optimally set at 64:36. Maintaining this ratio, UV‐10 and UV‐11 involved adjustments in the total concentrations, which unexpectedly led to a reduction in SI values. The decrease in UV‐10 was due to the lowered transmittance of both beneficial and harmful wavelengths, while in UV‐11, an increase in overall light transmittance was observed. These outcomes indicate that a balanced addition of TiO_2_ and FeO(OH)·H_2_O, near the 64:36 ratio, is favorable for sunscreen formulations. To enhance sun protection, integrating filters for harmful light can be efficacious, as demonstrated by UV‐12. This example showcases the incorporation of an organic UV filter into the optimized blend of TiO_2_ and FeO(OH)·H_2_O.

### 3.2 Cell Viability and Collagen I Level

**FIGURE 3:**
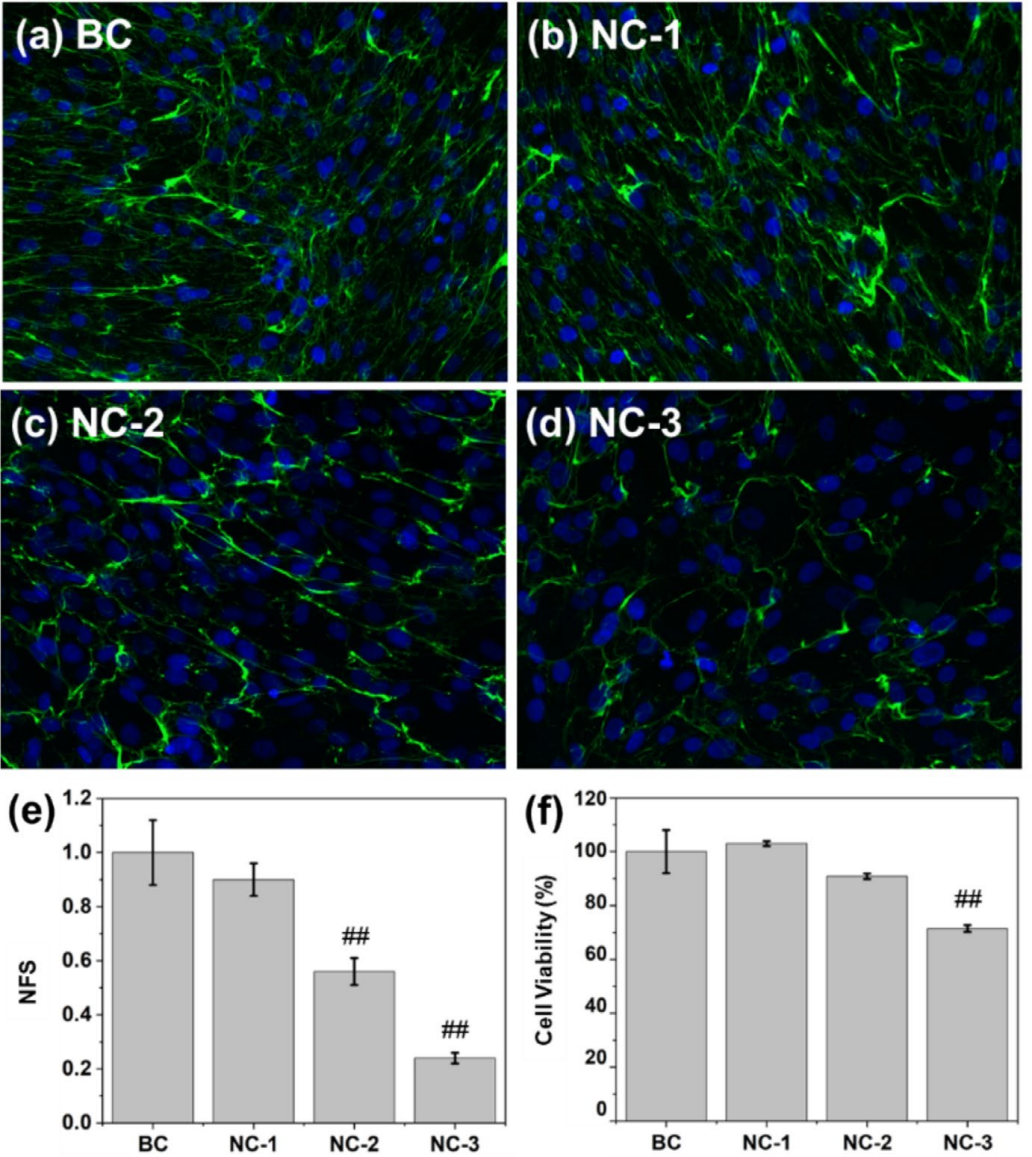
Cell viability and collagen I content under various light‐exposure conditions. (a–d) Immunofluorescence photos: Blue color—cell nuclei, green color—collagen I; (e, f) Statistical comparison.

Initially, the suitable light exposure condition for cell modeling was determined. In cases NC‐1 to ‐3, cells, unprotected by sunscreen, were subjected to various light intensities. Figure 3 illustrates a comparison of cell viabilities and collagen I levels following exposure. With respect to cell viability, the findings for NC‐1 and NC‐2 mirrored that of BC, whereas NC‐3 exhibited a notably lower result, indicating that the light exposure in NC was excessively intense, causing a significant amount of cell death. Upon comparing collagen I levels (NFS values) of NC‐1, NC‐2, and BC, declines were observed in both NC‐1 and NC‐2. However, no statistical difference was discernible between NC‐1 and BC. Consequently, the exposure condition of NC‐2 was determined to be the most appropriate and was adopted for cell modeling in subsequent tests.

**FIGURE 4:**
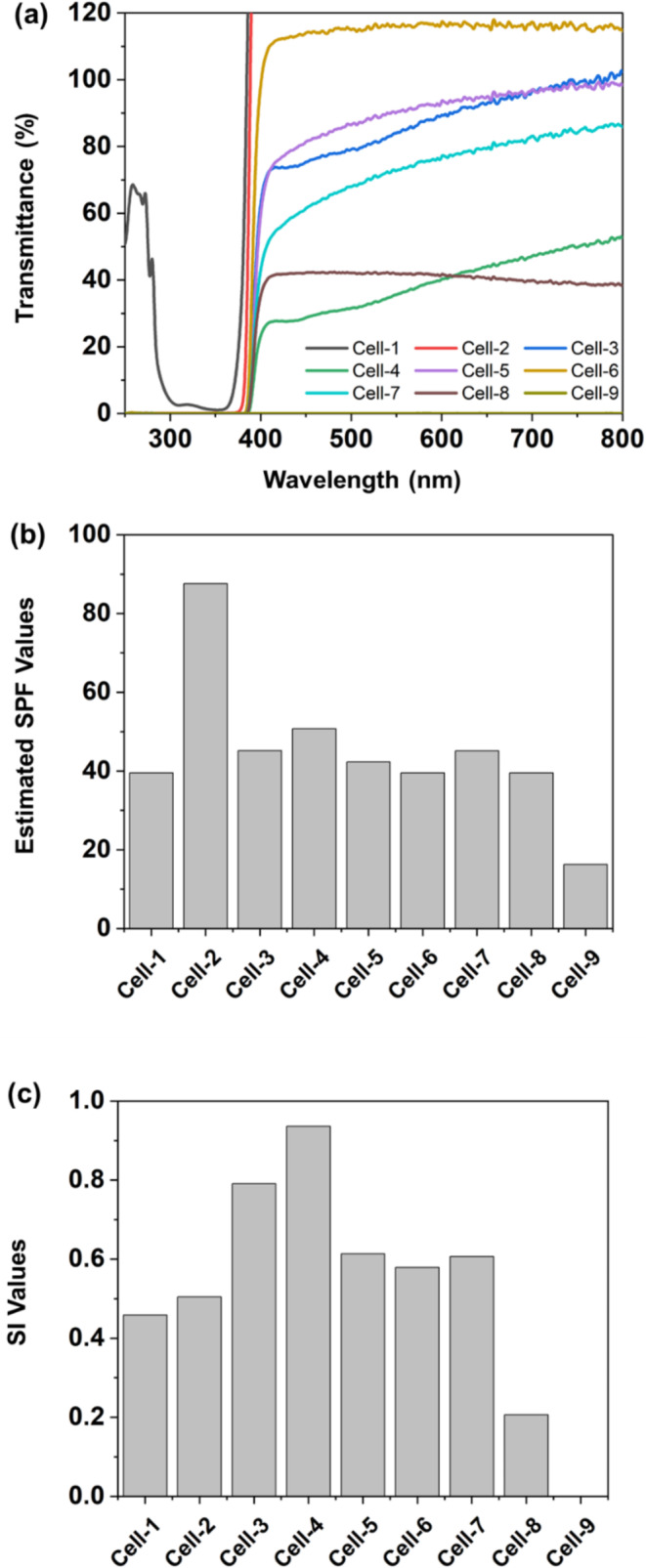
(a) UV–Vis transmittance spectrum, (b) estimated SPF values, and (c) SI values of different sunscreen formulas Cell‐1 to Cell‐9.

Under optimal model conditions, a variety of sunscreen formulations were subjected to cell experiments. Figure 4 presents the transmittance, estimated SPF values, and SI values for these formulations. Generally, the formulas show similar estimated SPF values ranging from 40 to 50, with the notable exceptions being Cell‐2, which contains a high level of UV filter, and Cell‐9, which solely comprises ZnO. However, their transmittance characteristics and SI values vary significantly. Formulas like Cell‐1 and Cell‐2, which contain only UV filters, achieved SI values of 0.46 and 0.50, respectively. In Cell‐3 and Cell‐4, the mixtures of TiO_2_ and FeO(OH)·H_2_O at the optimal ratio were added to the UV sunscreens, resulting in a marked increase in SI values. Notably, Cell‐4 achieved the highest SI value of 0.94. For Cell‐5 to Cell‐8, non‐spectral selective inorganic sunscreens were added in addition to UV filters, which led to a significant decrease in SI values compared to Cell‐3 and Cell‐4. Cell‐9, containing only ZnO, shows an SI value as low as 0.001.

In summary, these formulas were designed to explore various combinations, including those with “high‐SPF, mid‐SI” like Cell‐2, “mid‐SPF, high‐SI” like Cell‐4, “mid‐SPF, low‐SI” like Cell‐8, and “low‐SPF, low‐SI” like Cell‐9.

**FIGURE 5:**
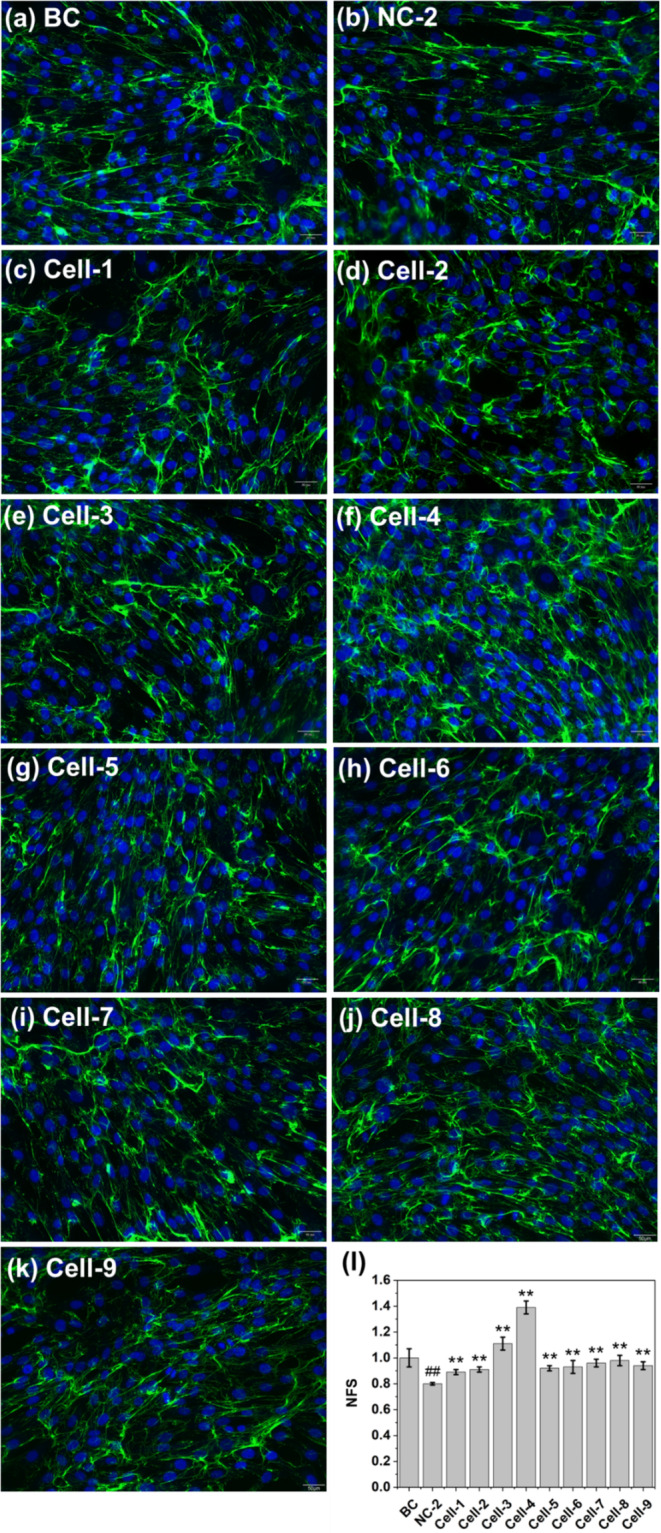
Collagen I contents after light exposure (280–760 nm, 10 J/cm^2^). (a–k) Immunofluorescence photos: Blue color—cell nuclei, green color—collagen I; (I) Statistical comparison.

Figure 5 presents a comparison of collagen I content among the different formulas. As anticipated, Cell‐4 demonstrated the highest collagen I content, which can be attributed to its spectral selectivity. Specifically, in Cell‐1 and Cell‐2, although there was a reduction in collagen I levels compared to the blank control (BC), the levels were significantly higher than that of negative control (NC‐2), indicating that conventional sunscreens have a protective effect on collagen. Nonetheless, this protective effect appears to have its limits; even when the concentration of organic filters in Cell‐2 was doubled in comparison to Cell‐1, there was no notable change in collagen levels. In Cell‐3 and ‐4, where the formulas included the optimal ratio mixture of TiO_2_ and FeO(OH)·H_2_O, there was a significant increase in collagen I content, with levels that even surpassed the BC results. By contrast, in Cells‐5 to ‐9, there was no discernible difference in collagen levels compared to Cell‐1. It be noted that Cell‐9, despite having a low SPF, maintains the Collagen I level parity to the cases with high SPF values, implying that low SPF value was sufficient for collagen protection in this cellular model.

## 4 Discussion

**FIGURE 6:**
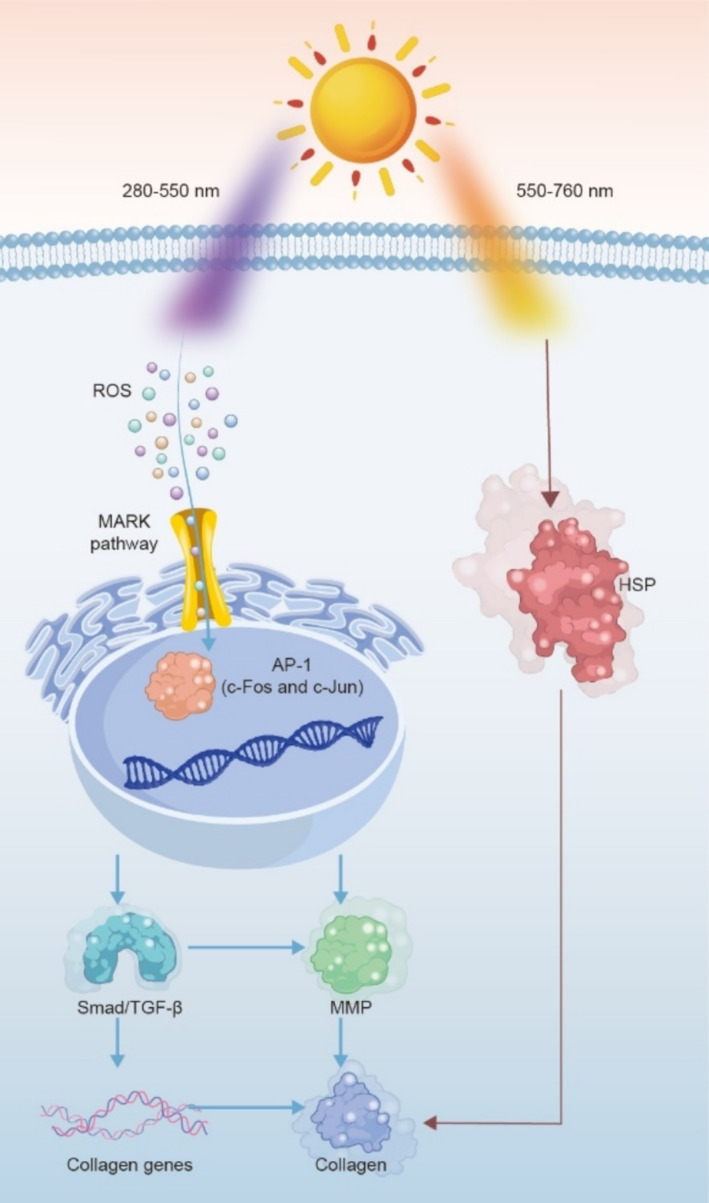
Degradative and promotive effects of sunlight on collagen synthesis.

The influence of sunlight encompasses both inhibitory and stimulative effects on skin collagen. As shown in Figure 6, the mechanism of inhibition primarily operates through cellular oxidative metabolism mediated by ROS primarily induced by high‐energy light [21]. ROS can drive the synthesis of matrix metalloproteinases (MMPs) by inciting the mitogen‐activated protein kinase (MAPK) and activating the activator protein 1 (AP‐1) heterodimer composed of c‐Fos and c‐Jun. Further, MMPs can break down collagen and elastin in the ECM subsequently, directly diminishing the content of collagen [22]. Concurrently, AP‐1 can down‐regulate the expression of the transforming growth factor‐β (TGF‐β) type II receptor. This leads to a disruption in the downstream Smad/TGF‐β signaling pathway and a decrease in the transcription of collagen I genes encoding type I collagen precursors [23], indirectly lessening the biosynthesis of collagen.

On the other hand, collagen promotion is primarily facilitated by a process involving limited thermal injury repair. Visible light with long wavelengths, also known as yellow and red light, has a significant photothermal effect when it acts on the skin tissue, inducing cells to express protective heat shock proteins (HSPs) [24]. When cells are exposed to heat or other stimuli, the increased synthesis of HSPs can act as molecular chaperones and bind with denatured proteins [25], playing a role in maintaining protein homeostasis by repairing or accelerating the degradation of misfolded proteins. Meanwhile, HSPs within cells have a protective effect on cell apoptosis caused by heat, oxidative stress, and other factors [26], inhibiting the generation of stress‐activated protein kinases, proteolytic enzymes, and oxygen‐free radicals and obstructing p53‐mediated cell apoptosis. Despite the spectral selectivity of the sunscreen employed, it cannot fully counteract sunlight's inhibitory effect on collagen. Nonetheless, its stimulative effect outweighs the inhibitory influence evidently.

However, it is essential to point out that with the increase of light energy, or with the increase in the proportion of UV and HEV in the light energy distribution, as can occur in high‐altitude locales, seaside areas, or at midday during summer, the deleterious impact of harmful light on collagen will be markedly intensified [27, 28]. Such conditions would in turn compromise the efficacy of spectral selective sunscreen. In these scenarios, the inclusion of organic filters capable of absorbing wavelengths below 550 nm into the formulation could boost the filtering effect against detrimental lights.

Moreover, this study only validated the efficacy of the spectral selective sunscreen at the cellular level, without considering the influence of skin tones on its effectiveness. Some researchers have found type II, III, and higher skin Fitzpatrick skin model is more vulnerable to hyperpigmentation induced by HEV and UVA [29] but is naturally better protected against UVB, due to the downstream pathway of Opsin‐3, tyrosinase, and dopachrome tautomerase, predominantly developed in the melanocytes of individuals with dark skin [30]. Therefore, Southeast Asian people with principally type II skin [31] are prone to impact by HEV and more suitable for spectral selective sunscreens in daily life.

## 5 Conclusion

The optimal combination of TiO2 and FeO(OH)·H_2_O has been found to exhibit remarkable spectral selectivity in sunlight filtration. This formula is capable of absorbing harmful wavelengths in the range of 280–550 nm and allowing the transmission of beneficial wavelengths within the 550–760 nm range. When integrated with conventional UV filters, this formula presents a positive effect on collagen I synthesis in cellular experiments. These findings pave the way for a promising approach in the development of future sunscreen formulas, potentially offering additional benefits to skin health.

## Author Contributions

X.Z.; X.W.; Y.W.; and Y.C. carried out the experiments and analyzed the data. X.Z.; F.J.; and Z.L., wrote the manuscript.

## Conflicts of Interest

The authors declare no conflicts of interest.

## Data Availability

The data that support the findings of this study are available on request from the corresponding author. The data are not publicly available due to privacy or ethical restrictions.
